# W′ expenditure and reconstitution during severe intensity constant power exercise: mechanistic insight into the determinants of W′

**DOI:** 10.14814/phy2.12856

**Published:** 2016-09-28

**Authors:** Ryan M. Broxterman, Phillip F. Skiba, Jesse C. Craig, Samuel L. Wilcox, Carl J. Ade, Thomas J. Barstow

**Affiliations:** ^1^ Department of KinesiologyKansas State UniversityManhattanKansas; ^2^ Department of Anatomy and PhysiologyKansas State UniversityManhattanKansas; ^3^ Department of Sports MedicineAdvocate Lutheran General HospitalPark RidgeIllinois; ^4^ Department of Health and Exercise ScienceUniversity of OklahomaNormanOklahoma

**Keywords:** Critical power, exercise tolerance, modeling, W′

## Abstract

The sustainable duration of severe intensity exercise is well‐predicted by critical power (CP) and the curvature constant (W′). The development of the W′_BAL_ model allows for the pattern of W′ expenditure and reconstitution to be characterized and this model has been applied to intermittent exercise protocols. The purpose of this investigation was to assess the influence of relaxation phase duration and exercise intensity on W′ reconstitution during dynamic constant power severe intensity exercise. Six men (24.6 ± 0.9 years, height: 173.5 ± 1.9 cm, body mass: 78.9 ± 5.6 kg) performed severe intensity dynamic handgrip exercise to task failure using 50% and 20% duty cycles. The W′_BAL_ model was fit to each exercise test and the time constant for W′ reconstitution (*τ*_W′_) was determined. The *τ*_W′_ was significantly longer for the 50% duty cycle (1640 ± 262 sec) than the 20% duty cycle (863 ± 84 sec, *P* = 0.02). Additionally, the relationship between *τ*_W′_ and CP was well described as an exponential decay (*r* ^2^ = 0.90, *P* < 0.0001). In conclusion, the W′_BAL_ model is able to characterize the expenditure and reconstitution of W′ across the contraction–relaxation cycles comprising severe intensity constant power handgrip exercise. Moreover, the reconstitution of W′ during constant power severe intensity exercise is influenced by the relative exercise intensity, the duration of relaxation between contractions, and CP.

## Introduction

The relationship between power output and exercise tolerance within the severe intensity exercise domain is well described by a hyperbolic equation, establishing the critical power (CP) and the curvature constant (W′) (Monod and Scherrer 1965; Whipp et al. 1982; Poole 2008):

(1) $$t=\frac{W^{\prime }}{\left(P-\text{CP}\right)}$$

where *t* is the duration of exercise and *P* is the power output. CP is the power asymptote of this relationship and as such is the highest attainable steady state for aerobic energy production without continually utilizing W′, and thus, demarcates the heavy and severe intensity exercise domains (Moritani et al. 1981; Poole et al. 1988; Jones et al. 2008; Poole 2008; Copp et al. 2010; Vanhatalo et al. 2010; Dekerle et al. 2012; Broxterman et al. 2014, 2015a). W′ is the curvature constant of the power‐duration relationship and represents a finite capacity for exercise performed above CP, for which task failure ensues with the complete expenditure of W′ (Monod and Scherrer 1965; Miura et al. 1999, 2000; Coats et al. 2003; Fukuba et al. 2003; Ferguson et al. 2007).

W′ is expended when the power output exceeds CP and is reconstituted when the power output is below CP (Ferguson et al. 2007; Chidnok et al. 2012, 2013). The precise mechanisms determining W′ remain uncertain, but in healthy populations, are associated with intramuscular energy store depletion (Monod and Scherrer 1965; Miura et al. 1999, 2000; Jones et al. 2008) and fatigue‐inducing metabolite accumulation (Coats et al. 2003; Fukuba et al. 2003; Ferguson et al. 2007; Jones et al. 2008). Additionally, the mechanisms determining W′ are associated with factors that may constrain or arise from these aforementioned alterations in the myofiber milieu, such as the breadth of the severe intensity domain (Burnley and Jones 2007; Vanhatalo et al. 2010), the magnitude of the oxygen uptake slow component (Murgatroyd et al. 2011), and the development of fatigue (Vanhatalo et al. 2011; Broxterman et al. 2015b). Although the mechanisms which determine W′ are uncertain, it has previously been demonstrated that the reconstitution of W′ from a fully depleted state to a fully replenished state is curvilinear in nature (Ferguson et al. 2010).

Integrating the mechanistic underpinnings of CP and W′, Skiba et al. (2012) developed a mathematical model that permits the calculation of the remaining W′ (W′_BAL_) at any time during exercise:

(2) $$W_{\mathrm{BAL}}^{\prime }=W^{\prime }-\int_{0}^{t}W_{\exp}^{\prime }\cdot e^{\frac{-(t-u)}{\tau_{W^{\prime }}}}\cdot \text{du}$$

where W′_exp_ is the accumulating expended W′ (as *P* − CP), (*t* − *u*) is the time between segments of exercise where power output is less than CP, and *τ*_W′_ is the time constant of the reconstitution of the W′. This model built upon previous work (Morton and Billat 2004) by incorporating the curvilinear reconstitution of W′. The W′_BAL_ model has been validated and successfully utilized to characterize the expenditure and reconstitution of W′ during intermittent exercise protocols with interspersed bouts of exercise above and below CP (Chidnok et al. 2012; Skiba et al. 2012, 2014b). The reconstitution of W′ is influenced by the recovery power output (Chidnok et al. 2012; Skiba et al. 2012) and there is an inverse relationship between *τ*_W′_ and the difference between recovery power output and CP (Skiba et al. 2012). In a subsequent study, it was further demonstrated that the reconstitution of W′ is also influenced by the duration of each recovery phase, as decreasing the recovery duration from 20 sec to 5 sec increased *τ*_W′_ by ~58% (Skiba et al. 2014b). Application of the W′_BAL_ model to intermittent exercise protocols has provided mechanistic insight into the nature of W′, as well as practical benefits for performance prediction (Skiba et al. 2012, 2014a,b, 2015). From these studies, it is evident that the mechanisms determining *τ*_W′_ are directly influenced by recovery phase intensity and duration. However, it remains to be determined if the mechanisms determining *τ*_W′_ are also influenced by power output within the severe intensity domain.

Typical constant power exercise is actually comprised of intermittent muscle contraction–relaxation cycles (e.g., cycling and running). During these cycles, W′ expenditure would be confined to the muscle contraction phase (e.g., downstroke of cycling cadence and stance phase of running), but it is not known if W′ reconstitution occurs during the relaxation phase (e.g., upstroke of cycling cadence and flight phase of running). W′ reconstitution during these relaxation phases may explain the overall slightly curvilinear, rather than strictly linear, depletion of W′ previously demonstrated during constant power cycling exercise (Skiba et al. 2012, 2014b). However, it may also be that the relaxation phase duration during typical constant power exercise is insufficient to allow for relevant W′ reconstitution. Additionally, it is currently unknown if the influence of CP on *τ*_W′_ demonstrated for intermittent exercise protocols (Skiba et al. 2012), holds true during typical constant power exercise. Successful application of the W′_BAL_ model to individual bouts of typical constant power exercise will increase our understanding of the mechanism(s) determining W′ and exercise tolerance. Therefore, the purpose of this investigation was to assess the influence of relaxation phase duration and power output on W′ reconstitution during dynamic constant power severe intensity exercise. We tested the hypotheses that (1) the *τ*_W′_ would be shorter when exercise was performed with a longer relaxation phase duration, (2) the rate of reconstitution of W′ during the relaxation phases of constant power exercise would influence exercise duration, (3) *τ*_W′_ would be constant across power outputs within the severe intensity domain with the same relaxation cycle duration, and (4) there would be an inverse relationship between *τ*_W′_ and CP.

## Methods

### Experimental procedures

Modeling procedures were conducted on data previously collected in Broxterman et al. (2014). All experimental procedures were approved by the Institutional Review Board of Kansas State University and conformed to the standards set by the *Declaration of Helsinki*. Written informed consent was obtained after subjects were informed of the overall protocol and the potential risks of participation. Six men (24.6 ± 0.9 years, height: 173.5 ± 1.9 cm, body mass: 78.9 ± 5.6 kg) volunteered to participate in the study. Subjects were free of overt cardiovascular or metabolic disease, determined via medical health history evaluation. Testing sessions were separated by at least 24 h and the subjects were instructed to abstain from vigorous activity during the 24 h prior to testing.

**Figure 1:**
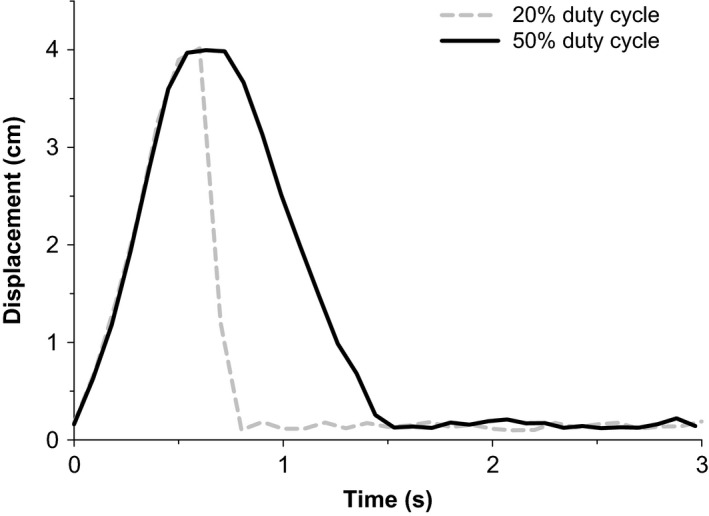
Displacement profiles for the 50% and 20% duty cycles in a representative subject. A 1.5 sec contraction phase and a 1.5 sec relaxation phase comprised the 50% duty cycle, while a 0.6 sec contraction phase and a 2.4 sec relaxation phase comprised the 20% duty cycle. Both duty cycles had a concentric contraction phase duration of 0.6 sec.

Testing was conducted using a custom‐built two‐handed handgrip ergometer, which was calibrated prior to the study. During each test, subjects were seated in front of the ergometer and grasped the handrail with both forearms approximately at heart level. Severe intensity constant power exercise was performed using a 50% duty cycle (1.5 sec contraction: 1.5 sec relaxation) and a 20% duty cycle (0.6 sec contraction: 2.4 sec relaxation) at a frequency of 20 contractions min^−1^ and a fixed displacement of 4.0 cm. The concentric contraction duration was held constant at 0.6 sec and the contraction–relaxation cycle total duration at 3.0 sec for both duty cycles (Fig. 1). An audio recording with the specific timing was used in conjunction with feedback provided by an investigator to ensure correct timing. Testing sessions were continued until task‐failure (*T*_lim_), determined as the inability to successfully complete the requisite 4 cm displacement for three consecutive contraction cycles. Peak power (*P*_peak_) was determined for each duty cycle using an incremental test initiated at 1.0 W and increased by 0.5 W min^−1^ until task‐failure. The constant power exercise tests were performed at power outputs eliciting exhaustion between 2 and 15 min and the data from these tests were fit with eq. (1) for the determination of a duty cycle specific CP and W′.

### Modeling analyses

Modeling analyses were individually conducted on three constant power output tests per duty cycle. For each subject, the three constant power tests per duty cycle were rank ordered by intensity as high, middle, and low. Modeling analyses were additionally conducted on tests performed at the same difference between the power output (*P*) and CP for both duty cycles, such that W′ expenditure per contraction would be similar per duty cycle for each subject (CP_diff_). The data from these tests were fit by the W′_BAL_ model (eq. (2)) by inputting the number of Joules expended above CP per contraction, and the *τ*_W′_ was varied iteratively until the W′_BAL_ = 0 J at *T*_lim_. Additionally, these data were fit by eq. (3):

(3) $$W_{\mathrm{BAL}}^{\prime }=W^{\prime }-\int_{0}^{t}W_{\exp}^{\prime }$$

to provide a predicted *T*_lim_ without allowing for W′ reconstitution to influence exercise duration. An estimate of the contribution of the relaxation phase W′ reconstitution to exercise tolerance was quantified by the difference between the actual *T*_lim_ and the predicted *T*_lim_ using eq. (3).

### Statistical analyses

Two‐way repeated measures ANOVA (duty cycle × intensity) were used to compare the *T*_lim_ and *τ*_W′_ data for the low, middle, high, and CP_diff_ intensities. Tukey's post hoc analyses were conducted when significant main effects were detected. Student's paired *t*‐tests were used to compare the predicted *T*_lim_ to the actual *T*_lim_ for each intensity within each duty cycle. The relationship between the mean *τ*_W′_ and mean CP across both duty cycles was assessed using regression analysis. Statistical significance was accepted at *P* ≤ 0.05. Date are presented as mean ± SEM.

## Results

The 20% duty cycle *P*_peak_ (7.4 ± 0.2 W) and CP (5.3 ± 0.2 W) were significantly greater than the 50% duty cycle *P*_peak_ (6.2 ± 0.2 W, *P* < 0.001) and CP (4.1 ± 0.2 W, *P* < 0.001), whereas W′ was not significantly different between the 20% duty cycle (479 ± 45 J) and the 50% duty cycle (477 ± 56 J, *P* = 0.95).

**Figure 2:**
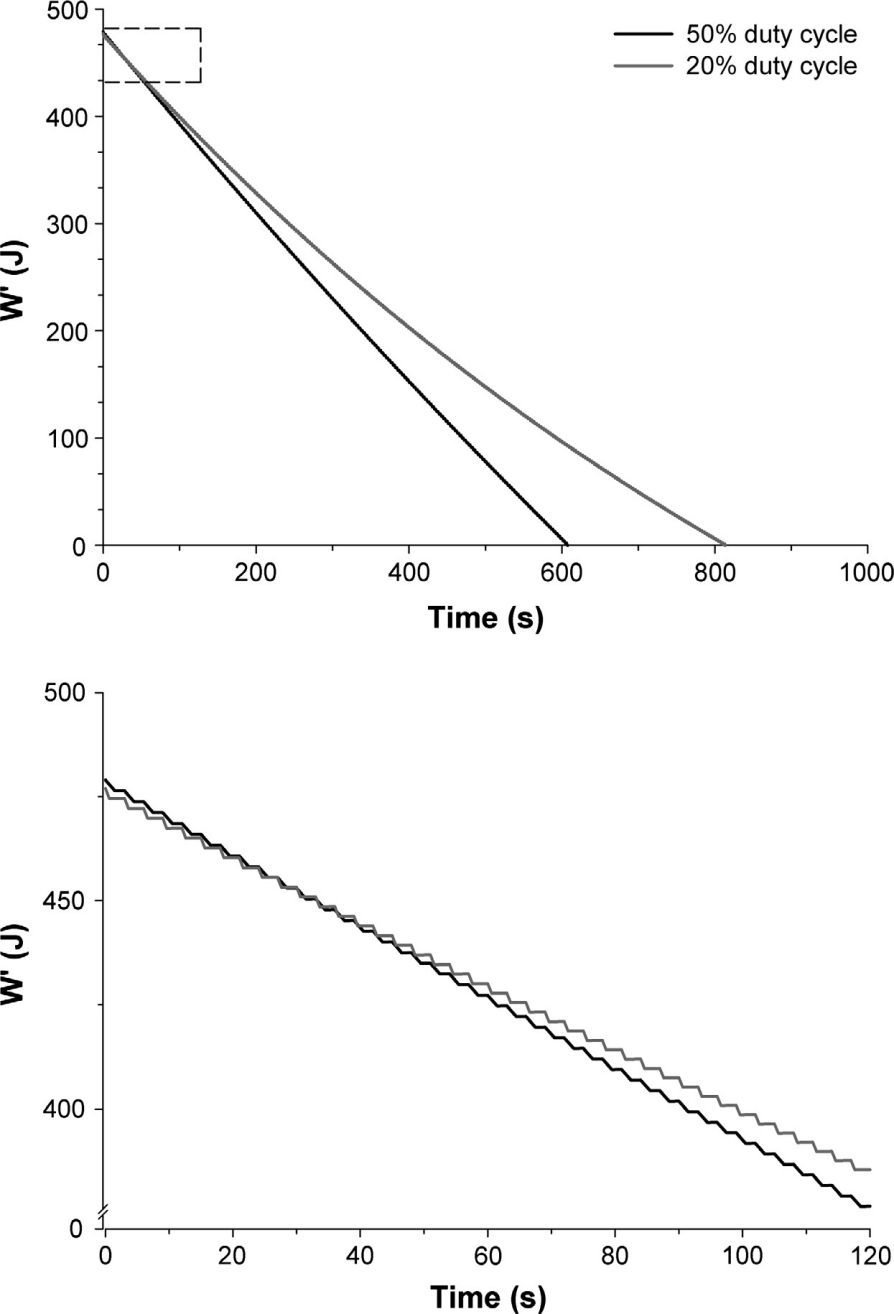
W′_BAL_ model during constant power exercise at a similar CP_diff_ for both the 50% and 20% duty cycles in a representative subject. *Top panel:* W′_BAL_ model across the entire duration of exercise. *Bottom panel:* W′_BAL_ model across the initial 120 sec. The difference between the power output and critical power (CP_diff_) was 0.87 W and 0.80 W for the 50% and 20% duty cycles, respectively. The box in top panel indicates the portion that is enlarged in the bottom panel.

**Figure 3:**
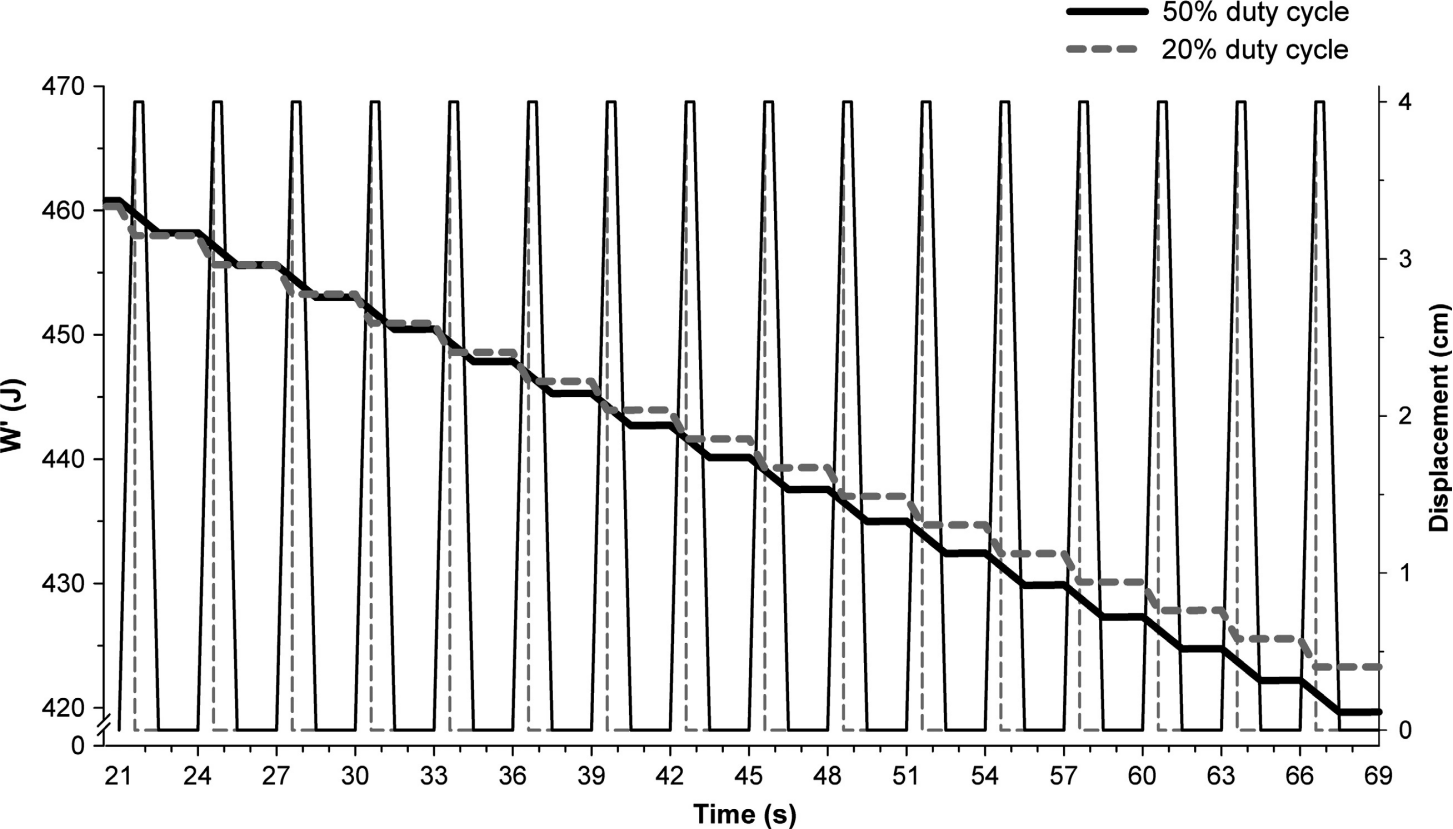
W′_BAL_ model and displacement during constant power exercise at a similar CP_diff_ for both the 50% and 20% duty cycles in a representative subject. W′ (*thick lines*) and displacement (*thin lines*) data are depicted across twelve contraction–relaxation cycles to show detail. The difference between the power output and critical power (CP_diff_) was 0.87 W and 0.80 W for the 50% and 20% duty cycles, respectively.

**Figure 4:**
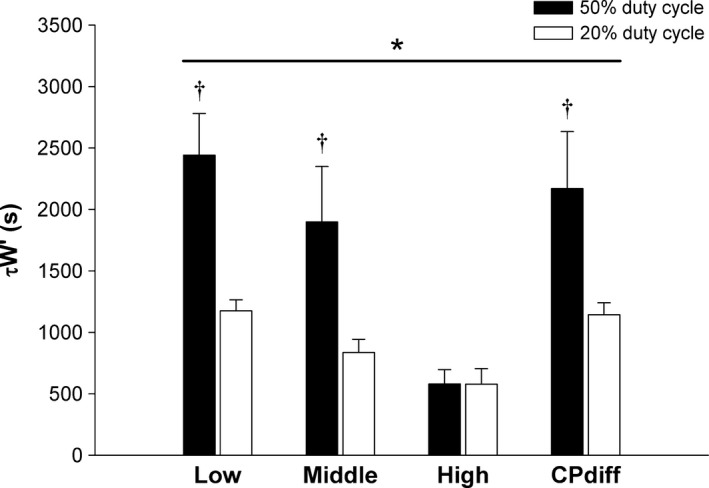
W′_BAL_ derived *τ*_W′_ for the 50% and 20% duty cycles. Calculated time constant for W′ reconstitution (*τ*_W′_) from the W′_BAL_ model applied to constant power exercise tests performed at low, middle, and high intensities, and similar differences between the power output and critical power (CP_diff_) for both duty cycles.*Significant effect of duty cycle (*P* = 0.03) and intensity (*P* < 0.001), as well as an interaction effect (*P* = 0.02). †Significantly different (*P* < 0.05) from 20% duty cycle data.

The dynamic reconstitution and utilization of W′ with the W′_BAL_ model are depicted in Figures 2 and 3. There were no significant differences between intensities across the two duty cycles at the low (50% duty cycle: 83 ± 3%*P*_peak_ and 20% duty cycle: 85 ± 4%*P*_peak_), middle (50% duty cycle: 92 ± 3%*P*_peak_ and 20% duty cycle: 93 ± 2%*P*_peak_), and high intensities (50% duty cycle: 108 ± 1%*P*_peak_ and 20% duty cycle: 108 ± 2%*P*_peak_), whereas each intensity was significantly different from the others within each duty cycle. The *T*_lim_ was significantly longer for the 20% duty cycle than the 50% duty cycle for the low intensity (50% duty cycle: 639 ± 58 sec and 20% duty cycle: 754 ± 82 sec, *P* < 0.05), but there were no significant differences between duty cycles at the high (50% duty cycle: 224 ± 12 sec and 20% duty cycle: 226 ± 26 sec) and middle intensities (50% duty cycle: 360 ± 32 sec and 20% duty cycle: 383 ± 27 sec). For *τ*_W′_, significant effects of duty cycle (*P* = 0.03) and intensity were detected (*P* < 0.001), as well as a significant interaction (*P* = 0.02). The average *τ*_W′_ was significantly longer for the 50% duty cycle (1772 ± 228 sec) than the 20% duty cycle (933 ± 71 sec, *P* = 0.03). Specifically, the *τ*_W′_ was significantly longer for the 50% duty cycle than the 20% duty cycle at the low (50% duty cycle: 2442 ± 337 sec and 20% duty cycle: 1175 ± 90 sec, *P* = 0.003) and middle intensities (50% duty cycle: 1899 ± 451 sec and 20% duty cycle: 836 ± 108 sec, *P* = 0.01), but not significantly different at the high intensity (50% duty cycle: 579 ± 117 sec and 20% duty cycle: 578 ± 127 sec, *P* = 0.99) (Fig. 4). Additionally, a significant exponential decay relationship was detected between *τ*_W′_ for each duty cycle and the corresponding CP (*r* ^2^ = 0.90, *P* < 0.0001) (Fig. 6).

**Figure 5:**
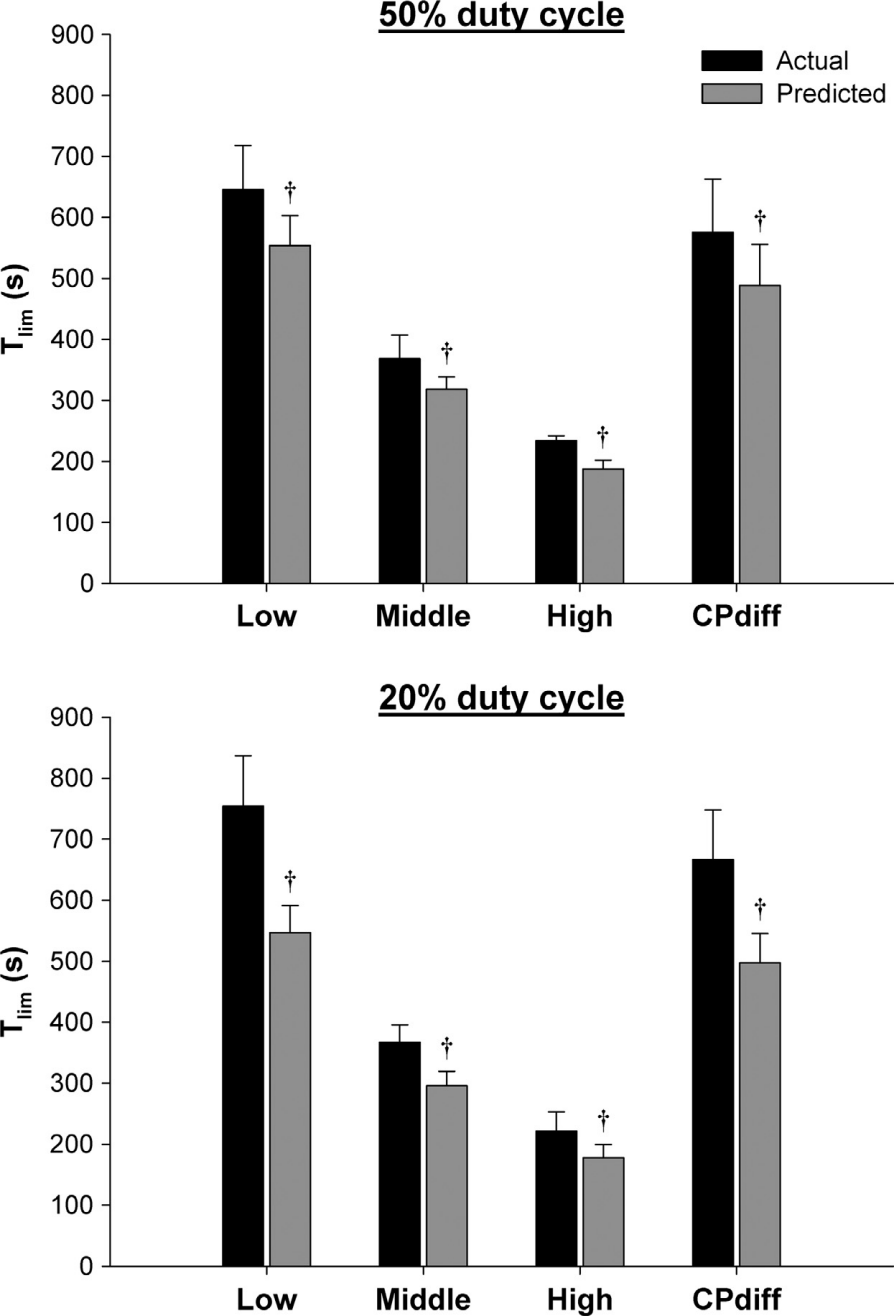
Influence of W′ reconstitution on exercise tolerance. Time to task‐failure (*T*_lim_) data for the actual constant power tests and predicted *T*_lim_ data modeled without allowing for W′ reconstitution at low, middle, and high intensities and similar differences between the power output and critical power (CP_diff_) for both duty cycles. †Significantly different (*P* < 0.05) from actual *T*_lim_ within the intensity.

The predicted *T*_lim_ without allowing for W′ reconstitution was significantly shorter within the 50% duty cycle for the low (554 ± 49 sec vs. 645 ± 73 sec, *P* = 0.02), middle (319 ± 19 sec vs. 369 ± 39, *P* < 0.05), and high intensities (188 ± 15 sec vs. 234 ± 8 sec, *P* = 0.001) and within the 20% duty cycle for the low (547 ± 44 sec vs. 754 ± 82 sec, *P* = 0.004), middle (296 ± 24 sec vs. 367 ± 28 sec, *P* < 0.001), and high intensities (178 ± 13 sec vs. 221 ± 32 sec, *P* = 0.02) (Fig. 5).

The CP_diff_ was not significantly different between the 20% duty cycle and the 50% duty cycle (1.0 ± 0.1 W vs. 1.1 ± 0.2 W, *P* = 0.40), whereas the absolute power output was significantly greater for the 20% duty cycle (6.3 ± 0.1 W) than the 50% duty cycle (5.1 ± 0.2 W, *P* < 0.001) due to the differences in CP between duty cycles. *T*_lim_ was not significantly different between the 20% duty cycle (666 ± 37 sec) and the 50% duty cycle (575 ± 88 sec *P* = 0.06), as W′ expenditure per contraction was not significantly different as either absolute (20% duty cycle: 3.1 ± 0.1 vs. 50% duty cycle: 3.2 ± 0.5 J, *P* = 0.40) or relative (20% duty cycle: 0.6 ± 0.1 vs. 50% duty cycle: 0.7 ± 0.1%W′, *P* = 0.50) values. However, the *τ*_W′_ was significantly shorter for the 20% duty cycle (1142 ± 405 sec) than the 50% duty cycle (2170 ± 363 sec, *P* = 0.01) (Fig. 4, Table 1). In addition, the predicted *T*_lim_ was significantly shorter than the actual *T*_lim_ for both the 50% duty cycle (489 ± 67 sec vs. 575 ± 88 sec, *P* = 0.03) and the 20% duty cycle (498 ± 48 sec vs. 666 ± 82 sec, *P* = 0.006) (Fig. 5).

**Table 1:** W′_BAL_ model derived time constants of W′ reconstitution (*τ*_W′_) in seconds

Subject	Low		Middle		High	
	50% duty cycle	20% duty cycle	50% duty cycle	20% duty cycle	50% duty cycle	20% duty cycle
1	1981	820	914	555	528	1179
2	3835	1225	3620	704	297	565
3	2308	1504	2266	1310	781	493
4	2033	1140	2573	729	220	322
5	2953	1224	1043	778	968	549
6	1541	1136	975	940	682	361
Mean	2442	1175^a^	1899	836^a^	579	578
SEM	337	90	451	108	117	127

a Significantly different (*P* < 0.05) from 50% duty cycle.

## Discussion

The purpose of this investigation was to assess the influence of relaxation phase duration and power output within the severe intensity domain on W′ reconstitution during dynamic exercise. It was demonstrated that *τ*_W′_ was shorter when exercise was performed with a longer relaxation phase duration, in agreement with our first hypothesis. It was also demonstrated that the W′ reconstitution during the relaxation phases between contractions, in aggregate, prolonged exercise tolerance compared to if reconstitution had not occurred, consistent with our second hypothesis. Inconsistent with our third hypothesis, it was demonstrated that *τ*_W′_ was not constant across exercise intensity. Finally, it was demonstrated that the *τ*_W′_ and CP were inversely correlated, as predicted by our fourth hypothesis.

### Influence of relaxation duration on *τ*_W′_

The contraction–relaxation cycles that comprise dynamic constant power exercise reflect the typical intermittent exercise protocol, merely on a smaller time scale. Applying the W′_BAL_ model to constant power exercise allowed for the pattern of W′ expenditure and reconstitution to be characterized within a contraction–relaxation cycle. In this study, the relaxation phase duration was longer for the 20% duty cycle (2.4 sec) than the 50% duty cycle (1.5 sec). The *τ*_W′_ was significantly longer for the 50% duty cycle than the 20% duty cycle for the low and middle intensities, but was not significantly different at the high intensity. Additionally, the modeling analyses were conducted on power outputs with the same difference between power output (*P*) and CP (CP_diff_) for both duty cycles, such that the W′ expenditure per contraction was similar and any differences in *τ*_W′_ would be observed without additional confounding influences. Again, it was demonstrated that *τ*_W′_ was shorter for the 20% duty cycle than the 50% duty cycle.

It is important to highlight that *τ*_W′_ represents the time‐course of W′ reconstitution and not the magnitude. It seems logical that a greater magnitude of W′ reconstitution would occur for the 20% duty cycle than the 50% duty cycle due to the longer relaxation phase duration. However, the shorter *τ*_W′_ demonstrates a faster W′ reconstitution for the 20% duty cycle than the 50% duty cycle. We have previously demonstrated that blood flow and oxygen extraction attain greater values for the 20% duty cycle than for the 50% duty cycle (Broxterman et al. 2014). Thus, the enhanced time‐course of W′ reconstitution in the 20% duty cycle may be the result of the increased blood flow and oxygen extraction speeding the recovery of depleted energy stores, the removal of fatigue‐inducing metabolites, and the reversal of fatigue.

The importance of the W′ reconstitution during the relaxation phases on exercise duration was assessed by modeling the data without allowing W′ reconstitution. If W′ reconstitution had not occurred during the relaxation phases, exercise duration would have been reduced by ~15% on average for the 50% duty cycle and ~24% for the 20% duty cycle. It has previously been demonstrated that the fall in W′ during constant power cycling exercise is slightly curvilinear, rather than strictly linear (Skiba et al. 2012, 2014b). Rather than a mathematical oddity of the W′_BAL_ model, the present investigation suggests that W′ reconstitution occurs during the relaxation phase (i.e., upstroke of pedal cadence during cycling). This finding offers additional context for other studies. For example, it has been demonstrated that the 3‐min all‐out test to assess CP and W′ is sensitive to pedal cadence (Vanhatalo et al. 2008). Altering cadence would be expected to alter the time for W′ reconstitution and therefore the amount of work performed above CP, precisely as observed by Vanhatalo et al. (2008). This evidence suggests that despite the apparently miniscule W′ reconstitution during each relaxation phase in isolation, the cumulative reconstitution of W′ across all of the relaxation phases during constant power exercise serves to prolong exercise duration.

### W′ reconstitution during constant power exercise

A greater magnitude of W′ utilization per muscle contraction occurs as power output increases above CP. Interestingly, the current data demonstrate that the rate of W′ reconstitution between contractions also increases as power output increases above CP. However, for exercise intensities above CP, the balance between W′ utilization and reconstitution is such that a net W′ utilization per muscle contraction–relaxation cycle occurs and W′ is utilized throughout the exercise until it is depleted and the requisite power output can no longer be maintained. The increase in the rate of W′ reconstitution as a function of exercise power output was more pronounced in the 50% duty cycle than the 20% duty cycle, such that the differences between duty cycles at the low and middle exercise intensities were not present at the high exercise intensity. These findings suggest that, in addition to the relaxation phase duration, the mechanisms determining *τ*_W′_ are also influenced by the power output within the severe intensity domain.

In this study, it was also demonstrated that *τ*_W′_ was inversely related to CP. As CP represents the highest steady state for oxidative metabolism, this relationship suggests that the physiological variables associated with oxidative metabolism (e.g., mitochondrial function and blood flow) also influence the mechanisms determining *τ*_W′_ (e.g., the rate of PCr resynthesis and the removal of fatigue inducing metabolites). It is also apparent from this relationship that a maximal rate of W′ reconstitution may exist. The inverse relationship between *τ*_W′_ and CP in this study is consistent with previous findings for intermittent exercise protocols of a shorter *τ*_W′_ for longer recovery phase durations (Skiba et al. 2014b) and an inverse relationship between *τ*_W′_ and recovery power output (Skiba et al. 2012). Consistent with the findings from intermittent exercise (Skiba et al. 2012), the relationship between *τ*_W′_ and CP (recovery power output in previous studies) was best described by an exponential regression.

### Methodological considerations

**Figure 6:**
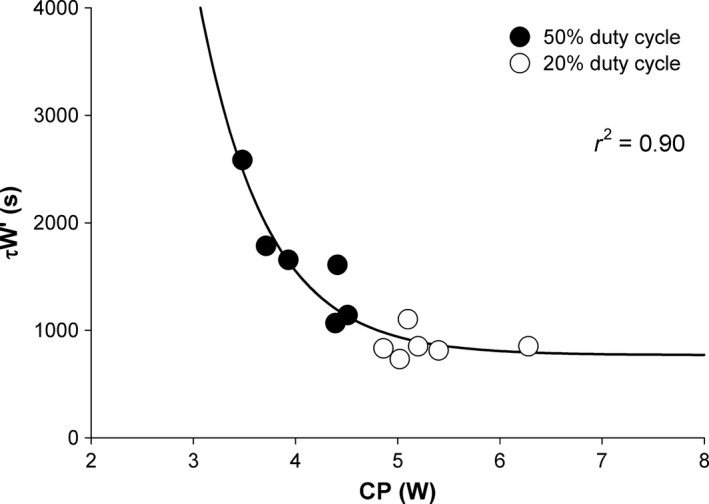
Relationship between *τ*_W′_ and CP for both 50% and 20% duty cycles. Mean time constant for W′ reconstitution (*τ*_W′_) versus the mean CP across both duty cycles.

In the W′_BAL_ model, it was assumed that W′ expenditure began immediately when the power output exceeded CP, W′ reconstitution began immediately when the power output decreased below CP, and W′ recovered with an exponential time course, which were based on evidence from previous investigations (Ferguson et al. 2010; Skiba et al. 2012). A significant exponential relationship was detected between *τ*_W_ and CP (Fig. 6). However, it should be noted that this relationship may also be well‐described by a linear fit. Future studies with a greater spread in CP values are needed to more confidently characterize this relationship. In this study, it was demonstrated that despite the relatively short relaxation durations between contractions, W′ reconstitution was sufficient to prolong exercise duration. While this evidence suggests that during dynamic exercise W′ reconstitution occurs, it is necessary to investigate the influence of exercise modality on W′ reconstitution. The handgrip exercise utilized in this study consisted of relatively longer relaxation durations (1.5 or 2.5 sec) compared to typical cycling or running (e.g., 60 rpm ≈ 0.5 sec relaxation). It remains to be determined if a minimum relaxation duration exists, below which the W′ reconstitution is no longer influenced or is negligible with regard to contraction duration. If these findings translate to more common exercise modalities such as cycling or running, these results may indicate that the “true” W′ may be overestimated from typical determination of CP and W′, as the reconstitution of W′ is not accounted for in the constant power tests. However, any overestimation of W′ would likely be consistent within a given exercise modality, as the variables that influence the relaxation phase duration (i.e., pedal cadence) are typically controlled or the participant performs each exercise test in a similar manner. Thus, even if an overestimation of the absolute “true” value of W′ occurs, the accuracy of predicting the time to task‐failure remains (Ferguson et al. 2010).

### Perspectives and significance

The findings of the current investigation provide important insight regarding the mechanisms determining W′. The current data demonstrate that any mechanism(s) (in isolation or in combination) purported to determine W′ must display a discernible oscillatory (contraction–relaxation) pattern with a relatively fast time‐course (such as shown by Chung et al. 1998 for phosphocreatine breakdown/resynthesis). Furthermore, the current data demonstrate that the mechanism(s) determining W′ are influenced by the contraction–relaxation cycle characteristics such as blood flow, oxygen delivery, and oxygen utilization. This evidence that the contraction–relaxation cycle characteristics influence W′ expenditure and reconstitution provides valuable insight for interventions aimed at enhancing exercise tolerance across the health spectrum from elite athletes to patient populations. Interventions that are able to optimize oxygen delivery and utilization, relaxation phase duration, and recovery phase intensity will spare the expenditure of W′ (which is limited in capacity) and enhance exercise tolerance. Additionally, this greater understanding of how W′ is depleted during constant power exercise will increase the practical applicability of the power–duration relationship by allowing for enhanced activity strategies, such as optimizing cadence and breathing patterns, in athletic and patient populations.

## Conclusion

The current investigation applied the W′_BAL_ model across the contraction–relaxation cycles during dynamic exercise to characterize the expenditure and reconstitution of W′. It was demonstrated that the reconstitution of W′ was influenced by the power output within the severe intensity domain, the duration of the relaxation phase, and CP. Moreover, the W′ reconstitution during the relaxation phases between contractions, in aggregate, prolonged exercise tolerance compared to if reconstitution had not occurred. The current investigation demonstrates that for severe intensity constant power exercise (i.e., above CP) the reconstitution of W′ during the relaxation phases was insufficient to fully replenish the W′ expended during the contraction phases. Therefore, as the exercise continued a net depletion of W′ occurred and when W′ was fully depleted task failure ensued.

## Conflicts of interest

The authors report no competing interests for this work.
